# Use of Machine Learning in Interactive Cybersecurity and Network Education

**DOI:** 10.3390/s23062977

**Published:** 2023-03-09

**Authors:** Neil Loftus, Husnu S. Narman

**Affiliations:** Department of Computer Sciences and Electrical Engineering, Marshall University, Huntington, WV 25755, USA

**Keywords:** cybersecurity, machine learning, education

## Abstract

Cybersecurity is a complex subject for students to pursue. Hands-on online learning through labs and simulations can help students become more familiar with the subject at security classes to pursue cybersecurity education. There are several online tools and simulation platforms for cybersecurity education. However, those platforms need more constructive feedback mechanisms, and customizable hands-on exercises for users, or they oversimplify or misrepresent the content. In this paper, we aim to develop a platform for cybersecurity education that can be used either with a user interface or command line and provide auto constructive feedback for command line practices. Moreover, the platform currently has nine levels to practice for different subjects of networking and cybersecurity and a customizable level to create a customized network structure to test. The difficulty of objectives increases at each level. Moreover, an automatic feedback mechanism is developed by using a machine learning model to warn users about their typographical errors while using the command line to practice. A trial was performed with students completing a survey before and after using the application to test the effects of auto-feedback on users’ understanding of the subjects and engagement with the application. The machine learning-based version of the application has a net increase in the user ratings of almost every survey field, such as user-friendliness and overall experience.

## 1. Introduction

Cybersecurity can be difficult for students to pursue in college, like many other STEM fields, while there are many factors that contribute to this, one of the primary reasons for this is the lack of exposure in high school. In 2022, only slightly over 50% of public schools had computer science education [1]. This lack of pre-college exposure is a major driver of inequity in computer science [2]. Research has shown that computer sciences have a notably higher rate of drop-out or major switching compared to other STEM majors [3]. However, research has also shown that high school computer science education is a predictor of success in higher education, especially among women, who are notably under-represented in the current field of computer science [4]. While not a replacement for formal education, interactive tools can be used to ease students into the subject, as well as allow teachers who are not explicitly trained in computer science to convey concepts to students [5].

One way to assist with computer science cybersecurity education while also keeping the program free and accessible is through open educational resources (OER). All OER resources are available to be used in an education context with no purchase or permission needed [6]. One type of resource that works exceedingly well as an OER is interactive visual simulations. Once produced, these simulations can be used repeatedly by any amount of users to learn about the subjects represented in the simulation.

Previously, we created an interactive program known as “the cybersecurity packet control simulator” (CSPCS) to teach students about the subjects of cybersecurity, internet working, and data structures [7,8]. The CSPCS application is based on previous research into the use of visual tools for computer science education, which showed that 54% of students preferred learning about data structures with an interactive augmented reality (AR) program, as opposed to more traditional methods [8]. Both of these studies are based on the concept of gamification, which is the act of turning education concepts into a game format. Research has shown gamification to be effective and preferred by students in an educational context [9,10].

Moreover, the CSPCS application without an auto-feedback version has been tested with a group of university students and is generally well received, with students having a 45% increase in self-evaluated understanding of the topics after using the program, as well as the overall experience rating averaging out to 3.67/5 [7]. The primary issues users have with the CSPCS application without an auto-feedback are with the user interface and explanation of concepts. Based on the feedback we received, we believe the level of abstraction between the user’s input and the simulation is detrimental to understanding, while we believe that making the input for the simulation more true to form would improve students’ understanding of the topics, this also brings several challenges. Using a more realistic system of input would drastically increase the technical knowledge required to use the application since that system of input would be text-based. This means that any small mistake made by the user would cause the input to fail. One way to increase realism while maintaining the ease of use would be to implement an automatic correction system.

While corrective feedback of any kind is known to assist students in learning, research has shown that “personalized feedback” correlates with helpfulness [11]. In [11], a number of different feedback modes were tested with students. The results show that digital feedback modes that were assessed as personalized were also assessed as more helpful, such as electronic annotations. Faculty resources are often limited, preventing instructors from being able to always provide manually personalized feedback. However, machine learning technologies can be used to replicate some facets of personalized feedback, such as informing the user what specific portion they are mistaken on.

There are other online platforms to explain cybersecurity and internetworking subjects. One of the well-known tools for cybersecurity and internetworking education is Cisco Systems Networking Academy (CSNA) [12]. CSNA is a more advanced platform that has beginner to advanced classes that are led by instructors in-person or in an online environment. CSNA is incredibly comprehensive, with hours of coursework; however, due to this, it can be overwhelming for less-experienced users. However, due to its complexity, it is not ideal for high school or inexperienced university students, who may find it difficult to approach. With this project, we aim to develop a tool primarily catered to students. The scope of this application is designed such that it can be used to help students visualize the core idea before continuing with more comprehensive instruction.

The objective of this paper is to develop a machine learning-based auto-feedback system over our previously developed application [7]. The key contributions of this paper is (i) the implementation of the application based on auto-feedback and console features—https://pws-cspcs-preview.herokuapp.com/ (accessed on 22 November 2022), (ii) the development of the machine learning model to provide feedback, (iii) carrying on field testing, and (iv) comparing the machine learning-based feedback (ML) and non-feedback (non-ML) versions based on our gathered data and users’ feedback. The main way we implemented this auto-feedback mechanism has been to improve the quality and realism of the input system. The increase in complexity caused by the text input system can be mitigated by using a system to correct user mistakes. This is performed by using machine learning (ML) in the application. If the user inputs an invalid command into the application, that command is then given to the ML model, which predicts what command the user most likely intended. This, combined with several manual feedback systems, provides the user with guidance upon making a common mistake, allowing them to have a more realistic experience while still being intuitive and easy to use.

The trial results of the ML version compare favourably over the non-ML version, with the most notable data point being an increase in the average overall experience (3.7 out of 5 for the non-ML version and 4.1 out of 5 for the ML version). It should be acknowledged that this trial has been performed primarily with second-year and beyond university students, and the program could be expanded to be more suitable for different audiences, such as high school students.

The rest of the paper is organized as follows: Section 2 describes the development process of the application. Section 3 describes the selection of the Machine Learning model. Section 4 explains the implementation of the application, as well as the machine learning development and implementation. Section 5 compares the results of the trial of the non-ML version of the application compared to the ML version. Section 6 discusses our explanations and comments on the results. Finally, Section 7 presents concluding remarks with future works.

## 2. Application Development

### 2.1. Background

The application discussed in this paper is an updated version of an application developed to teach late high school and early university students about cybersecurity and internetworking concepts. The application provides users with a series of levels where the user would control how bundles of information or “packets” were routed between devices in a simulated network. These levels would have the user learn about topics such as routing, weighted graphs, algorithms, pinging, and a cybersecurity attack known as the “man in the middle” [13,14,15,16,17,18,19,20,21]. All of these levels remain in the second version which we called as ML version of the application and were improved to more intuitively convey the ideas being taught.

### 2.2. Differences between the Non-Machine Learning and Machine Learning Versions

The non-machine learning (non-ML) version of the application uses Unity 3D for the framework and WebGL for web support [22,23,24,25]. This website is publicly hosted using Heroku [26,27]. Unity is a popular tool often used for game development, but it also has usage in animation and simulations. Its innate support for web browsers through WebGL build support made Unity ideal [22,23,24,25]. Additionally, Unity supports mobile without having to completely rebuild the project, allowing for easy continuation of this project. The non-ML version of this application is designed to convey the ideas of cybersecurity and routing, but to streamline the system of input to simple buttons. For the machine learning (ML) version, we decided to move away from simplified input to make the simulation more realistic. Because of this, we decided to make the new system of input the default, while still giving the user the option to enable the old input system if they prefer it. For this paper, we will only be discussing the functionality of the new command line system of input. Details about the button-based system of input can be found in [7].

### 2.3. Terminal Input

The system of input for the ML version is modelled as similar to the Windows command line. As such, the user controls the simulation almost completely through text input, with only a few UI elements were added when necessary. Changes had to be made to the currently existing level to make them compatible with this new input system. Primarily, the levels are set up so the home device and 127.0.0.1 is always the device sending packets since the terminal input is supposed to represent the command line of one specific computer, while the user input system is based on a publicly available tutorial, the internal processing and integration with the ML model is a custom implementation and is discussed in detail in Section 4.

## 3. Machine Learning Algorithm Selection

### 3.1. Python Model Analysis

An important step of this project was to evaluate multiple different forms of machine learning models and decide which would be optimal for this implementation. Previous research has been performed in which different types of machine learning models are concurrently compared. In an example, Ref. [28] by Modaresi et al. compared several different types of machine learning for predicting inflow into the Karhkeh reservoir. Similarly, for this paper, we test a number of different machine learning algorithms for our dataset of available commands and possible typos. The first are “logistic regression” and “Gaussian naive Bayes (NB)”, which fit data to a curve. The next models are support vector classifiers (SVC), which is a method that uses a line, plane, or even hyperplane to bisect and classify data. This includes “kernalized SVC” and “linear SVC”. “K-neighbours” classifies data by distance from other data points. “Perceptron” and “multi-layer perceptron (MLP)” are artificial neural networks (ANN), which are composed of several input and output layers that allow for prediction [28]. Finally, “matching” is a non-machine learning technique that uses Levenshtein distance to find the possible matching.

### 3.2. Analysis Methodology

To perform this analysis, we developed two programs that are based on the sci-kit learn library and its implementation of the algorithms mentioned in Section 3.1 [29]. The main difference between the two programs is the use of optimization according to feature extractions from the dataset. This dataset is composed of scrambled and misspelled words, with the labels being the correctly spelled version of those words, as explained in Section 4.5. For example, “msg” is the label, and “mgsasa” is the randomly generated typo based on the label. In the optimized version, Levenshtein distance, the unique number of characters, and the rate of matched character count in each position are used for feature extraction.

### 3.3. Analysis Results

**Figure 1:**
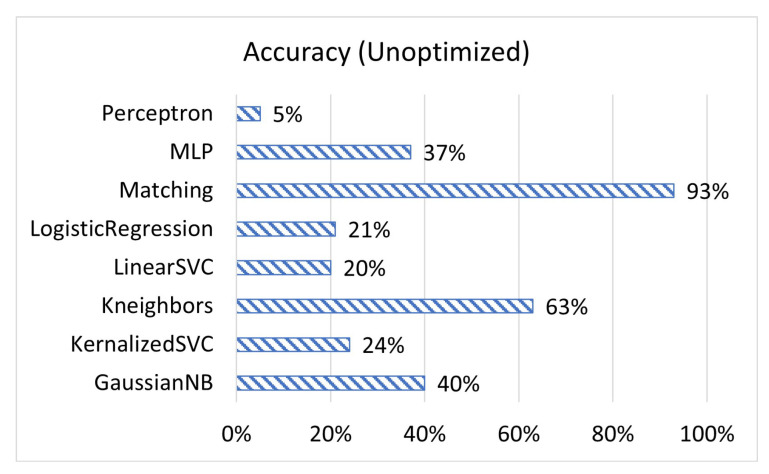
The accuracy of ML prediction compared to actual values for unoptimized models.

**Figure 2:**
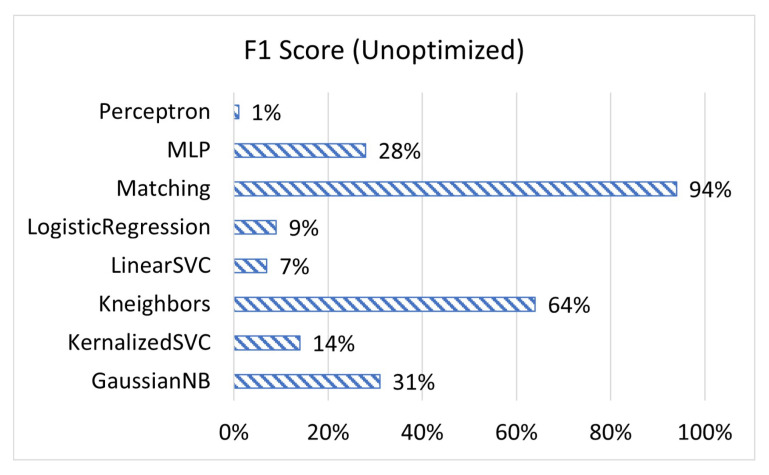
The F1 score of unoptimized models.

**Figure 3:**
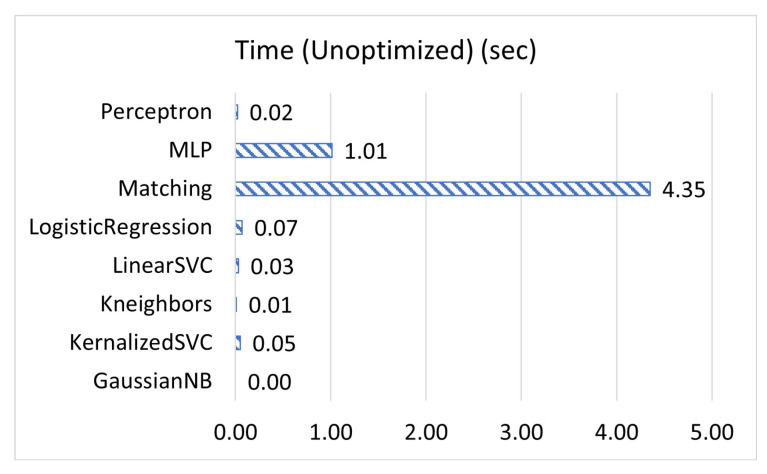
The time of prediction for unoptimized models, in seconds.

**Figure 4:**
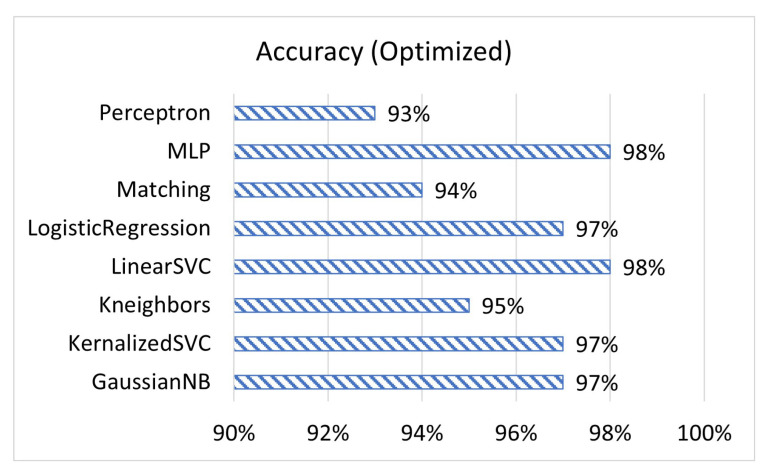
The accuracy of ML prediction compared to actual values for optimized models.

**Figure 5:**
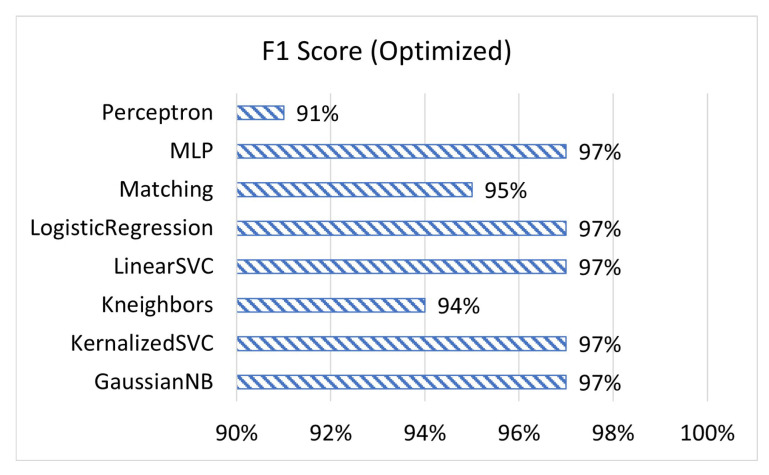
The F1 score of optimized models.

**Figure 6:**
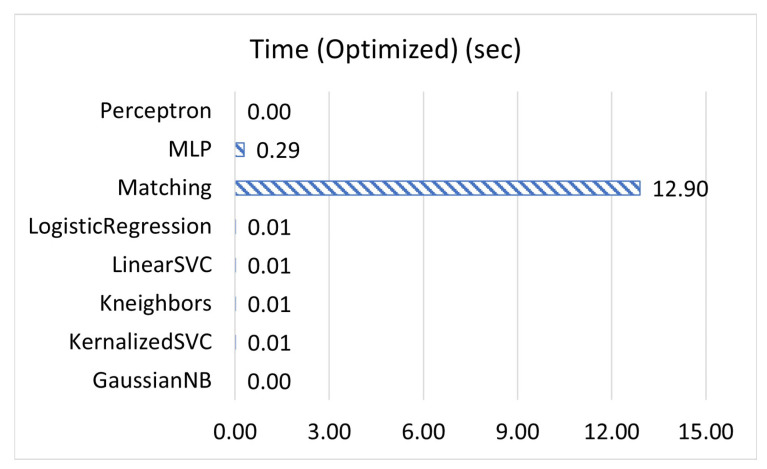
The time of prediction for optimized models, in seconds.

Figure 1, Figure 2 and Figure 3 display the accuracy, F1 score, and prediction time of the unoptimized models while Figure 4, Figure 5 and Figure 6 display the accuracy, F1 score, and prediction time of the optimized models.

Figure 1 and Figure 2 show that matching based on Levenshtein distance performs by far the best while unoptimized, while Figure 4 and Figure 5 show that all models performed well after optimization. It is important to note that K-neighbours shows the best performance compared to the other machine learning models in the unoptimized version. As shown in Figure 3 and Figure 6, the matching based on Levenshtein distance takes significantly more time.

Similarly, MLP takes significantly longer than all other forms of the machine learning models tested in both unoptimized and optimized versions because MLP is an ANN with several layers. Previous research has shown that while models such as K-neighbours may be more efficient for linear data, for more scattered data, ANNs often perform more accurately, with the converse being true as well [28]. Our data from these unit tests show that for the type of data, most types of machine learning models are able to perform adequately. In the end, the model was primarily selected due to language compatibility reasons.

### 3.4. TensorflowJS

While many powerful frameworks for machine learning exist, the chosen framework for this application is TensorflowJS [30]. As we are building upon a pre-established application, the machine learning model chosen would ideally be able to integrate into the application with minimal changes necessary. Since the application uses C# for Unity and Javascript (JS) for WebGL, it is necessary that the machine learning model be compatible with both languages [22,23,24,25]. Fortunately, Unity has the innate compatibility for communication between JS and C#, so as long as a JS-compatible machine learning model could be found, it would be integrated into the application. TensorflowJS is a JS spin-off of the popular and versatile Python machine learning library Tensorflow [31]. By using this framework, we would have access to the layer-based ML models of Tensorflow, which would allow us to train our own custom-made ML model [30]. This custom model is capable of reading a typographical error up to 10 characters long and predicting what command it is intended to be using pattern recognition.

## 4. Implementation

### 4.1. Resolution Improvements

One of the more impactful changes for user experience is the improvement in resolution and scaling. In the non-ML version, the application would only take up a portion of the website. However, through the use of a modified HTML template under the MIT license, the application scales dynamically to fit the size of the window [32].

### 4.2. Settings and Cookies

**Figure 7:**
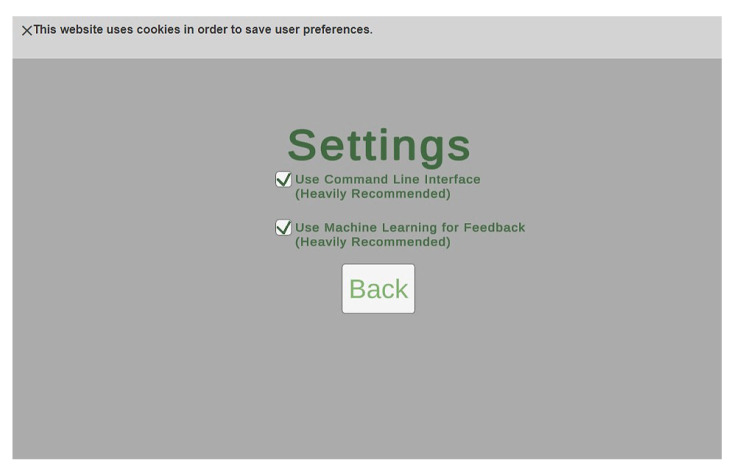
The settings page and cookies notification.

With the update to the input system, we want to provide users with an option to disable these features in case they prefer the old version of the application. This is performed through the use of the settings screen. Users can enable or disable both the command line interface and the machine learning system from the settings. Additionally, this application uses cookies to save the users’ settings if they leave the page [33,34]. To respect the privacy of the user, there is a banner notification to inform the user about the website’s usage of cookies, as shown in Figure 7.

### 4.3. Command Line and Tools

**Figure 8:**
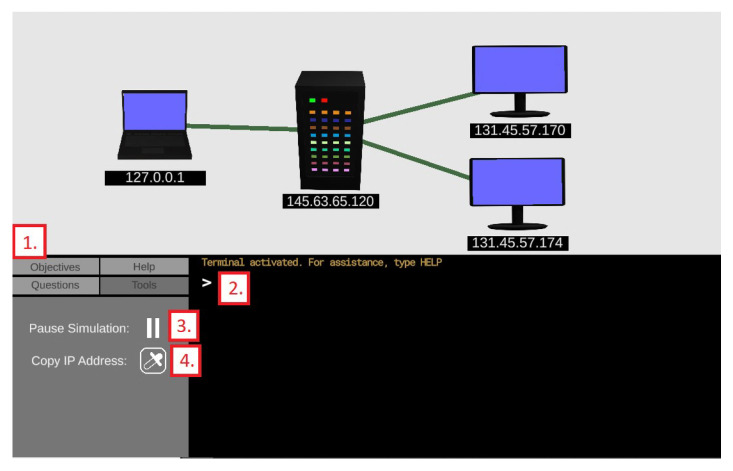
The command line interface and tools panel.

Figure 8 shows the implementation of the command line interface (CLI), as well as various tools for controlling the simulation. Relevant elements are marked with large red numbers. Element 1 is the selection panel, which allows the user to swap between the objective, question, help, and tools panels. With the exception of the tools panel, a new addition, the other three panels remain unchanged from the non-ML version. Element 2 is terminal. The user can select the terminal and type in a command to influence the simulation. The list of recognized commands and their functionality is explained in the next subsection. Element 3 is the pause button. This button freezes the simulation in place, allowing the user more time to evaluate what is occurring. Finally, element 4 is the eyedropper button. The user can click on this button and then click on a device in the simulation, and the IP address listed below that device will be copied into the terminal.

### 4.4. Text Processing and Tokenization

Before the user’s input can be processed by the program, it first needs to be tokenized. Tokenization is the process of breaking text into distinct words or phrases, known as tokens [35]. This is performed by identifying groups of characters as words or phrases and then identifying the meaning of those tokens. For this application, tokens are mostly words, so they can be detected by breaking the input string up by spaces. This allows most of the words to be easily isolated into tokens; however, because there are circumstances where more than one word can be put into a token, an exception must be added. The tokenizer ignores spaces that are between two quotes, meaning that any phrase in quotation marks is treated as a single token. For simplicity’s sake, the application is not case-sensitive, and will internally convert every letter to lowercase.

The next step is to discern the meaning of these tokens. The tokenizer accepts the input as a list of tokens and categorizes each token using a set of criteria. There are over 10 different categories of tokens, but all of these fall into one of three super categories: commands, variables, or errors. “Commands” represent the name of a terminal command. The tokenizer only recognizes the commands if they match the name exactly. Each of these commands represent a real Windows terminal command that is relevant for cybersecurity and networking. These commands represent the functionality of the actual command but are slightly modified to fit the introductory scope of the application. Some of the more complicated parameters of these commands are either simplified or removed. The commands utilized are: MSG, PING, TRACERT, ROUTE, PKTMON, CLS, and HELP [36]. MSG sends a packet containing a string message [37,38]. PING sends a packet which causes the destination device to return a packet back. TRACERT pings every device en route to an end destination. ROUTE displays the routing table of any given device. PKTMON shows a log of every packet a device has received. CLS empties the terminal of all text. HELP displays information about each of the commands. “Variables” are for processing miscellaneous data necessary for commands. This includes numbers, quotes, and IP addresses. Since there are several different values that can be considered valid input for these tokens, they each have specific criteria for identification. “Errors” are for handling incorrect inputs. There are two types of error tokens, unknown and invalid. A token is marked as unknown if the token cannot identify it as anything else. Any token that is marked as unknown, is first checked for specific errors, such as if it is a packet message that is not in quotes. Otherwise, it is sent to the machine learning model for prediction and automatic feedback. Invalid tokens occur when a token is recognized but does not meet the requirements for the command to execute. The most common example is if a valid IP address is provided, but there is no device that exists with that IP address.

The final step is syntax analysis after each token is recognized. In this step, the list of tokens is compared with the requirements for each command, to identify which command should be executed. A major challenge faced during the design step was how to handle optional parameters. Optional parameters affect a command’s functionality, but the command still executes if they are excluded. To address this, each parameter in a command’s requirements list is either marked as optional or mandatory. If a mandatory parameter is missing, the input is considered invalid for this command, and it moves to compare it with the next command. However, if the parameter is optional, the tokenizer continues to compare the input with the command’s requirements. If the input totally matches a command’s requirement list, it executes the code associated with that command. If not, it compares the input with the next command in the list. If the input matches none of the commands, it is marked as invalid, and an error is displayed.

### 4.5. Natural Language Processing

In the case that a token is marked as unknown and does not trigger any specific feedback, it is assumed to be a typographical error. In a normal command line, this would result in a generic error message. However, we provide specific feedback whenever possible. In order to provide this feedback, we use a specific type of machine learning known as natural language processing (NLP). NLP is specially designed for processing words in natural languages, such as English, and it can be used to find misspellings and typographical errors. To do this, we trained a custom TensorflowJS model to classify the type of commands. The first step in this process is to find the list of Windows commands and then select the ones appropriate for the application [36]. Next, we create our dataset. The type of machine learning employed in this project is known as “supervised learning”, which is categorized by its usage of labelled data for training. A dataset is labelled when there is also a label for each data point that categorizes it [39,40]. In this case, the data is the typographical error or misspelling of a command, and the label is the correct spelling. When the model is trained, it uses these labels and attempts to predict what label any given input corresponds to. For supervised learning to work, we needed to gather a large amount of labelled data. To accomplish this, we used a modified version of a typographical error-generating algorithm [41]. The main change we made to this algorithm was to modify it to prevent duplicate data from being generated.

Once the dataset is generated, we create a program to generate the machine learning model. Creating models in TensorflowJS is based on the Keras layers framework, which passes training data through several layers to improve results [42,43]. The model is primarily composed of long short-term memory (LSTM) layers. These LSTM layers are used for their ability to output a predicted sequence of data [44,45]. TensorflowJS is primarily designed to predict numerical data, particularly numerical data between zero and one. Because of this, we need to encode our text data into binary, using a method known as “one hot encoding”. This method turns each individual character into an array of binary values [46,47]. As we are working with character data, this conducted by having each character turned into an array of length equal to the number of recognized characters. Since we are using just the lowercase alphabet and blank space, this length is equal to 27. We are able to identify the character by checking what index the “one” is at, as every other value will be set to zero.

This process is repeated for each letter in the word and then repeated for each word in the dataset, making the input data into the model a three-dimensional binary array. Since LSTM returns a sequence, the output is also a three-dimensional binary array, but instead of being composed of 26 zeros and 1 one, it is instead composed of 27 values from zero to one. To convert this data to text, we reverse the encoding process with one key difference. Instead of identifying the letter by the index of the one in the array, we instead find the index of the largest value. When this process is complete, the model will have outputted a text sequence. The final step is to compare this sequence with the known commands. If the first few characters of any command match the sequence, it will be marked as a successful prediction, and it will return the predicted value. Otherwise, it will return “unknown”. The model uses sequence output instead of raw categorization in order to make false positives less common, as it will return “unknown” if it has no prediction it is confident enough in.

**Figure 9:**
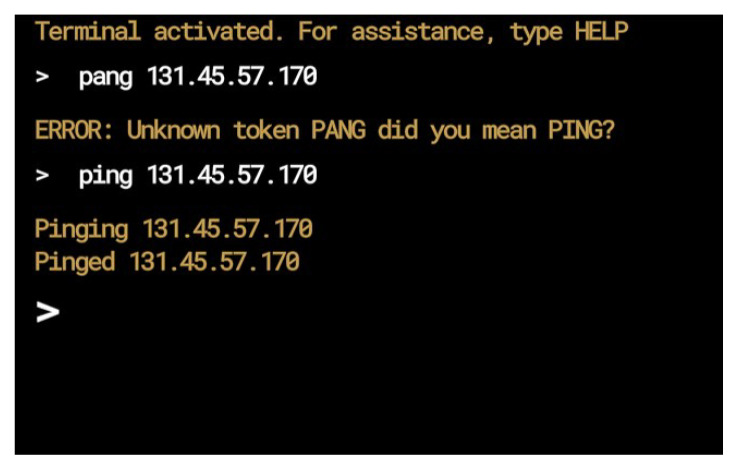
The machine learning system providing automatic feedback.

Once the model is finished training, it is exported to a file that can be quickly loaded by the website. Training a machine learning model can take upwards of several hours. By having the model pre-trained and exported to a file, the application can load and use it in mere seconds. With the machine learning model, the user’s mistakes are identified and feedback is provided without the need for an instructor, as shown in Figure 9.

## 5. Results

In order to measure the effectiveness of the program, the non-ML application was tested with a group of approximately 30 college students in computer science or computer science adjacent fields [7]. These students were tasked with completing a pre-survey, using the application, and then completing a post-survey. The questions asked of the pre-survey are as follows: student email, major/class rank, computer science section in which the survey was administered, self-described learning style (i.e., visual, kinetic), understanding on cybersecurity, computer networks, and routing on a scale from 1 to 5 (1 being the least, 5 being the most), and any other programs they had used to learn about similar topics. Students were then asked to complete the six implemented levels and fill out a post-survey after completing them. The questions asked in the post-survey were as follows: student email, major/class rank, computer science section, a short five question quiz on concepts taught in the application, a re-scoring of understanding from 1 to 5 on each concept covered, and then a set of qualitative questions about users’ opinions on the application. These qualitative questions covered: concepts that they thought were explained well and those that were not explained well, concepts that were interesting, understanding of the program’s purpose, opinions on user-friendliness, opinions on educational benefits, interest in using an improved version of the program, uniqueness compared to other programs, general suggestions, and overall opinion of the application [48,49].

All of these results from the non-ML application were evaluated and used to enhance the application [50]. After the application had been completed, a second trial was initiated to evaluate what impact the changes had on effectiveness. In order to make the data from the two trials more comparable, no questions from the original survey were changed, with the only difference being a few brief questions on the new features [48,49,51,52].

The comparison of user trials of the non-ML and ML versions of the application shows a net increase in almost every facet of the application, with user-friendliness and understanding of computer networks. The first quantitative metric that the two applications are compared on is the student’s self-rated understanding of the subjects being taught.

**Figure 10:**
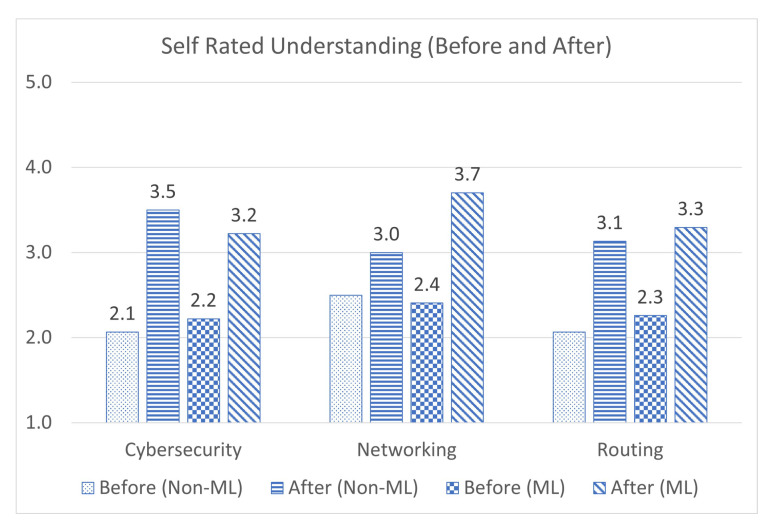
Comparison of self-rated understanding between two versions of the application.

The average final scores for understanding in the non-ML application were: cybersecurity (3.5/5), networking (3.0/5), and routing (3.1/5) [49]. In the ML version, the average scores were: cybersecurity (3.2/5), networking (3.7/5), and routing (3.3/5), as shown in Figure 10. These results demonstrate a slight decrease in cybersecurity understanding, a large increase in networking, and a slight increase in routing [52]. This seems counter-intuitive at first, as the ML version of the application increased the number of cybersecurity concepts. However, it is likely that there is a comparatively larger increase in networking concepts, causing students to view the application primarily as a tool for networking concepts. Additionally, the command line interface may have been more conducive to teaching networking concepts than cybersecurity.

**Figure 11:**
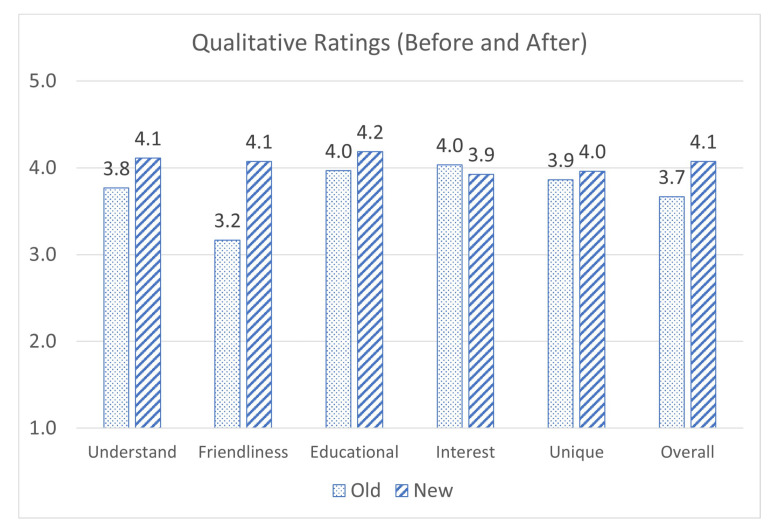
Comparision of review scores between two versions of the application.

Additionally, students were asked to rate the program from 1 to 5 on several qualitative metrics, as shown in Figure 11. Almost every one of these average metrics was improved in the trial of the ML application compared to the non-ML. Students’ average understanding of the program’s purpose increased from (3.8/5) to (4.1/5). The average user friendliness increased precipitously from (3.2/5) to (4.1/5). Students’ rating of the educational benefits increased from (4.0/5) to (4.2/5). The uniqueness of the program on average increased from (3.9/5) to (4.0/5). Finally, the overall rating increased from (3.7/5) to (4.1/5). The only metric that decreased was interest in using an improved version of the application from (4.0/5) to (3.9/5) [49,52]. The most notable increase was in user friendliness. We believe that the extra attention given to the UI, particularly the interactive terminal, accounted for this increase. Almost every category measured exceeded (4/5), with the only exception being interest in using the program again, meaning we have achieved our primary goal for a revision to the application. We believe that this decrease may have been due to the extended length, as it being longer may have resulted in users not feeling a need to use it any further after completing the whole thng.

**Figure 12:**
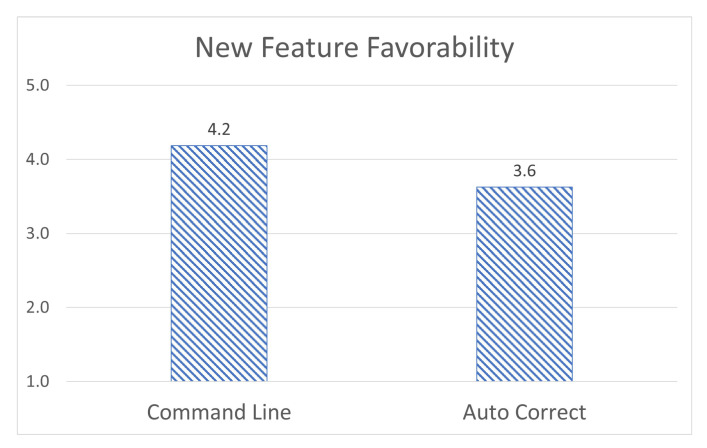
Evaluation of command line and auto-correction feature.

Finally, students were asked to rate their opinions on the new additions in this version of the application, primarily the text-based input and the machine learning-based corrections. Users rated their opinion on the text-based input as (4.2/5), and the helpfulness of the machine learning auto-correction as (3.6/5) [52], as shown in Figure 12. This shows while the text input system is widely appreciated, the auto-correct system is seen as less important. One rationale for this is that for users that did not make any mistakes in the text input system, the correct system would have been unnecessary for them. Additionally, the program is only trained to correct typographical errors and had no capacity to automatically detect syntax errors. We believe that a machine learning system that could correct and explain syntax errors would be drastically more helpful. We are planning to implement such functionalities in the future.

## 6. Discussion

While the surveys used for the trials were almost entirely unchanged, there were some possible imperfections that must be acknowledged. In the non-ML trial, a portion of the survey was focused on measuring the user’s self-described learning style and then comparing these results to the user’s results on a short content quiz. On further analysis, these metrics have some imperfections that make them less useful for evaluating the effectiveness of the program. The non-ML trial demonstrated no significant difference between user evaluation results correlating learning style. This is supported by recent research, which states the concept of distinct learning style categories that students clearly fall into is inaccurate [53]. Additionally, there are a few imperfections with the trial itself, primarily the number of questions and the questions themselves. Students were asked to answer five multiple-choice questions relating to the content presented in the application. Due to the low number of questions, a single incorrect answer would result in a decrease in that student’s score of 20%. This also meant that due to the relatively small sample size of around 30 testers, any student who did particularly poorly would heavily affect the average. In the second trial, the average evaluation score was around 70%, far lower than the 90% in the first trial. This decrease cannot solely be blamed on statistical outliers, however, as there are many possible explanations for the decrease. In order to properly compare evaluation results, the questions were unchanged between the two versions of the application. However, the ML version of the application is significantly expanded, with new concepts introduced and several of the old levels reworked. Because of this, the evaluation designed for the non-ML application is not optimal for the ML version [49,52]. Additionally, for brevity, users were only asked to complete five of the ten available levels, meaning it is possible that a user may have skipped a level that is asked about on the evaluation. This could have been remedied by specifically assigning five levels out of the ten that all users must complete.

## 7. Conclusions and Future Works

In this paper, we develop a platform for cybersecurity and networking education that can be used either with a user interface or command line and provide auto constructive feedback for the command line practices. We are partially interested in the observed users’ experiences on the machine learning-based auto-feedback version and non-machine learning version of the application. The field trials of the application demonstrated an increase in the majority of metrics covered by the survey, with a small portion of the metrics decreasing due to the survey not accounting for a shift in direction for the machine learning-based auto-feedback version of the application. The machine learning-based auto-feedback version of the application met the projected goal of increasing both the user-friendliness and overall experience to over (4.0/5) [52].

The primary goal of any future work on this project would be to improve user accessibility. Currently, the application is only available on computers that support WebGL, meaning that a person who only has a mobile device or an older computer cannot use the application. We believe that porting this application to mobile platforms would drastically improve the reach of the tool, especially amongst students who lack computers. For any future additions, we would likely include more guided instruction for the students in order to introduce students to the concepts before asking them to use the application. Our main approach to doing this would be to embed a brief training video on the application’s website, while this is primarily an interactive medium, we believe that some guided instruction may improve understanding, especially by clearing up common misunderstandings. Additionally, we plan to implement several accessibility changes, such as more language options or a mode for visually impaired or colour blind users. These changes would help accommodate students who may have traditionally been disadvantaged in computer science education.

## Data Availability

Not applicable.
